# Antihistamine response: a dynamically refined function at the host-tick interface

**DOI:** 10.1186/s13071-014-0491-9

**Published:** 2014-10-31

**Authors:** James J Valdés

**Affiliations:** Institute of Parasitology, Biology Centre of the Academy of Sciences of the Czech Republic, 37005 České Budějovice, Czech Republic

**Keywords:** Dynamics, Histamine, Lipocalin, Competitive binding, Tick saliva, Ticks

## Abstract

**Background:**

Ticks counteract host inflammatory responses by secreting proteins from their saliva that compete for histamine binding. Among these tick salivary proteins are lipocalins, antiparallel beta-barrel proteins that sequester small molecules. A tick salivary lipocalin has been structurally resolved and experimentally shown to efficiently compete for histamine with its native receptor (e.g., H1 histamine receptor). To date, molecular dynamics simulations focus on protein-protein and protein-ligand interactions, but there are currently no studies for simultaneous ligand exploration between two competing proteins.

**Methods:**

Aided by state-of-the-art, high-throughput computational methods, the current study simulated and analyzed the dynamics of competitive histamine binding at the tick-host interface using the available crystal structures of both the tick salivary lipocalin histamine-binding protein from *Rhipicephalus appendiculatus* and the human histamine receptor 1.

**Results:**

The attraction towards the tick salivary lipocalin seems to depend on the protonated (adding a hydrogen ion) state of histamine since the current study shows that as histamine becomes more protonated it increases its exploration for the tick salivary lipocalin. This implies that during tick feeding, histamine may need to be protonated for the tick salivary lipocalin to efficiently sequester it in order to counteract inflammation. Additionally, the beta-hairpin loops (at both ends of the tick salivary lipocalin barrel) were reported to have a functional role in sequestering histamine and the results in the current study concur and provide evidence for this hypothesis. These beta-hairpin loops of the tick salivary lipocalin possess more acidic residues than a structurally similar but functionally unrelated lipocalin from the butterfly, *Pieris brassicae*; comparative results indicate these acidic residues may be responsible for the ability of the tick lipocalin to out-compete the native (H1) receptor for histamine.

**Conclusions:**

Three explanatory types of data can be obtained from the current study: (i) the dynamics of multiple binding sites, (ii) competition between two proteins for a ligand, and (iii) the intrinsic molecular components involved in the competition. These data can provide further insight at the atomic level of the host-tick interface that cannot be experimentally determined. Additionally, the methods used in this study can be applied in rationally designing drugs.

**Electronic supplementary material:**

The online version of this article (doi:10.1186/s13071-014-0491-9) contains supplementary material, which is available to authorized users.

## Background

In response to tissue damage or a hypersensitivity reaction, histamine is released by mast cells and basophils that then bind to its native receptors (histamine receptors, H1R and/or H2R) to facilitate repairing agents or mediators of the immune response arriving at the injury site [1]. Salivary gland transcriptomes have revealed that ticks, as obligate hematophagous (blood feeding) arthropods, secrete many protein families at the injury site to counteract host physiological responses – antagonizing inflammation is one example of the mechanisms ticks employ. Among the major protein families discovered from tick salivary gland transcriptomes are lipocalins [2], antiparallel beta-barrel proteins that sequester small molecules. Several tick salivary lipocalins from these transcriptomes, e.g., from the tick *Ixodes persulcatus*, have been used in vaccinating mice showing a delay in the ability of ticks to imbibe a full blood meal [3]. Expressed sequence tag analyses of *Rhipicephalus (Boophilus) microplus* larvae also show that lipocalins are upregulated after feeding on *Babesia bovis* infected cattle [4].

Tick salivary lipocalins have been reported to sequester different host molecules that disrupt host physiological responses. For instance, a salivary lipocalin from the tick *Dermacentor reticulates* sequesters both serotonin and histamine causing an inhibition of guinea-pig ileum to contract [5]. A few salivary lipocalin histamine-binding proteins (HBPs) have been also reported from the tick *Rhipicephalus appendiculatus* (Ra-HBPs) [6]. The crystal structure of one of these tick salivary Ra-HBPs (Ra-HBP2) revealed a few structural deviations from the archetypal lipocalin protein family, that it sequesters two histamine molecules, and also causes an inhibition of guinea-pig ileum to contract by competing with H1R/H2R for histamine binding [6]. Tick lipocalins are also differentially expressed depending on the specific host they feed on, for instance among *Amblyomma* species [7]. Understanding the mechanics of tick salivary lipocalins will provide a better insight of the host-ectoparasite interface.

The advent of computational methods to simulate the molecular dynamics of protein-ligand interactions has advanced drug discovery and our understanding of molecular systems [8]. At the atomic level, molecular dynamics simulations have complemented experimental studies by simulating protein-ligand interactions at milliseconds timescales with high resolution [8] to describe, for example, the negative cooperativity of multiple ligands binding to cell surface receptors [9] or the protein conformational changes (inactive, intermediate and active state) of transmembrane G-protein coupled receptors (GPCRs) during protein-agonist interactions [10]. Experimental kinetic measurements on nitrophorin 7, a salivary lipocalin from the blood-sucking insect *Rhodnius prolixus*, show that disassociation of the native ligand (nitric oxide bound in the salivary glands of *R. prolixus*) at the host injury site causes an ‘open’ protein conformational change in order to sequester histamine [11] and that this change is controlled by the fluctuating pH at the host-ectoparasite interface [12]. Molecular dynamics studies on this nitrophorin indicate that the overall conformation of the beta-barrel is maintained with larger changes occurring at the beta-hairpin loops [11].

To date, no studies have computationally investigated the dynamic interactions occurring during the competition between two proteins for a ligand. This lack of knowledge leaves a gap on the fundamental molecular interactions of ligand pharmacological promiscuity and their targets. There still remain unanswered questions, for example, in the case of the tick salivary lipocalin, Ra-HBP2. Is histamine exploration of its native receptor (e.g., H1R) eliminated in the presence of the tick Ra-HBP2? What intrinsic molecular components cause histamine to possess a greater affinity for Ra-HBP2? Are there any specific residues on the surface of Ra-HBP2 responsible for histamine preference? Once bound to Ra-HBP2, how do interacting residues in proximity with histamine comport? Once Ra-HBP2 is fully saturated, will histamine explore its native receptor (e.g., H1R) more? Can more histamine potentially explore the double bound Ra-HBP2?

To answer these questions, the ready-made scripts from the state-of-the-art Protein Energy Landscape Exploration (PELE) server [13,14] were expanded on for simultaneous, unbiased ligand migration between two proteins. Landscape exploration is the mapping of protein conformational changes, energy and interacting molecules. Although PELE is not as robust as other molecular dynamics techniques that simulate global protein conformational changes, i.e., from inactive to active conformation [10,15], PELE can simulate the migration pathway of ligands and how residues interact in proximity of the ligand with minimal computational effort [13,14]. In this study, the PELE algorithm was used to simulate the competition of histamine binding between its native target, the human H1R, and the tick salivary lipocalin, Ra-HBP2.

## Methods

### Computer simulated protein preparation and refinement

Since x-ray crystallography cannot resolve hydrogen atoms for most protein structures the Schrodinger’s Maestro Protein Preparation Wizard [16] was used to assign hydrogen atoms and to optimize the assigned hydrogen-bond network for H1R (PDB: 3RZE) and Ra-HBP2 (PDB: 1QFT). A histamine molecule from PDB: 1QFT was also prepared separately in a similar fashion for each protonated state. Additionally prepared were the crystal structures of a tick Kunitz peptide (PDB: 2UUX) and a butterfly lipocalin (PDB: 1BBP), used as controls for competitive ligand binding simulations. The respective ligands for each protein structure were removed prior to preparation. After protein preparation an energy minimization – default conditions in the Schrodinger’s Maestro package – was performed on the crystal structures (including histamine) to alter the initial conformation and to remove any steric clashes prior to PELE simulations. Note that the human H1R (PDB: 3RZE) is the only histamine receptor with its structure resolved, therefore the only host receptor available. There is one missing region of the H1R (residues 167–175), the extracellular loop 2, that was modeled using the Prime application from the Schrodinger’s Maestro package [17].

### The PELE algorithm

A detailed explanation of the PELE algorithm can be found in references [13,14] and its many uses can be accessed at https://pele.bsc.es/. Briefly, PELE performs three steps. First, localized perturbations for the ligand are performed using random translations (tra_r) and rotations (rot_r). For the protein, perturbations are also performed using an anisotropic network model (ANM) [18] to drive the alpha-carbons to a new position after minimization. Secondly, PELE optimizes amino acid side chains in proximity to the ligand using a rotamer library [19] and steric filters. As a final step, PELE uses a truncated Newton minimizer and a surface generalized Born [20] implicit solvent for a minimization to achieve a local minimum after the initial perturbation. The PELE algorithm therefore performs, in an implicit solvent, localized perturbations then side chain sampling and finally relaxes the system conformation via minimization to simulate ligand migration and protein side chain-ligand interaction(s). This is repeated for a desired number of steps and can be performed in parallel using several computer-processing units (CPUs). The outcome is a series of trajectories (each step is a trajectory multiplied by the number of CPUs) representing conformational changes for side chains and ligand migration. A Monte Carlo Metropolis criterion implemented in the PELE algorithm either accepts or rejects these trajectories based on their calculated energies – if they are equal to/less than (accepts) or greater than (rejects) the initial calculated energy [13]. To calculate the energy, the PELE algorithm uses the optimized potentials for liquid simulations (OPLS-2005), a standard force field used to describe the potential energy of a molecular system [21]. Based on the OPLS force field, PELE calculates the energy of a molecular system as,

$$ E={E}_{AB}-\left({E}_A+{E}_B\right) $$

where the total energy (*E*) equals the receptor-ligand complex (*E*_*AB*_) minus the sum of its individual units for the receptor (*E*_*A*_; H1R or Ra-HBP2) and ligand (*E*_*B*_; histamine). The receptor-ligand complex (*E*_*AB*_) was used in this study to represent the energies of the complexes formed (e.g., H1R- or Ra-HBP2-histamine).

### Histamine migration simulations using PELE

The ready-made script for an unconstrained ligand binding site search provided by the PELE server (https://pele.bsc.es/) was used to analyze histamine migration for both individual (H1R or Ra-HBP2) and competitive binding (H1R and Ra-HBP2). The parameters for this script were slightly altered to increase the efficiency of the PELE algorithm (i.e., ~30% Monte Carlo accepted steps – a standard acceptance rate [14]). These alterations included increasing the number of steps (2000 steps) to provide sufficient sampling, increasing the area around the ligand for side chain perturbations (sprad 6.0) to reduce steric clashes, increasing ligand rotamer translations (tra_r 12.0) to allow histamine to move more freely, and randomizing side chains for unbiased local perturbations. The ANM option was the only parameter deleted for histamine migration since it was too computationally expensive for the PELE algorithm to calculate in the competitive binding simulations – possibly due to the presence of the two protein structures for histamine migration. Since H1R is in its inactive conformation and Ra-HBP2 is in an active conformation, the ANM was also deleted for individual binding simulations as a control to test whether PELE can sample the active site of both proteins without large protein conformational changes.

For competitive histamine binding a single PDB file was created using the initial coordinates that positions the tick Ra-HBP2 at a distance of 67 Ångstroms (Å) from the center of mass (COM) of the human H1R. All prepared histamine molecules were placed equidistant (42 Å) from the COM of both proteins as a starting point for each simulation. The control proteins (PDBs: 2UUX and 1BBP) were superpositioned onto the Ra-HBP2 coordinates to confirm unbiased histamine preference towards the orientation of Ra-HBP2. Note that either H1R or Ra-HBP2 was deleted from the same combined PDB file for individual histamine binding simulations. Out of the 48 CPUs running in parallel for the ligand migration simulations, there were a few CPUs that did not explore either protein (3–4 CPUs) exceeding a distance of 100 Å from their respective COMs, thus producing unfavorable binding energies ≥0 kcal/mol (as calculated by PELE). The scatter plots in this study are therefore represented at a cutoff distance of 100 Å and 0 kcal/mol for binding energies.

The PELE algorithm allows several tasks to be performed consecutively. For instance, as histamine reaches in close approximation (via unbiased ligand migration) to the protein active site (less than 10 Å of their respective COMs) the PELE algorithm can halt at that point then perform a second task to explore the active site more, known as spawning. Spawning allows the PELE algorithm to explore within a predetermined region (spawn point # xyz within 8.0 – the active sites of both proteins are ~8 Å from the COM). These two tasks (i.e., unbiased ligand migration and spawning) were included for the individual histamine binding simulations to show that (i) histamine can approach the active site of either protein in an unbiased and unconstrained fashion and (ii) can sufficiently sample the respective active site regardless of its inactive or active conformation.

### Induced-fit refinement

The reaction between a protein and ligand is initiated only after protein conformational changes caused by the ligand occur – this is known as induced-fit theory [22]. The PELE server (https://pele.bsc.es/) also provides a ready-made script for induced-fit refinement that was used for refining the position of the second histamine of the tick Ra-HBP2. The alterations made for the induced-fit refinement script to increase the efficiency of the PELE algorithm were increasing the number of steps (2000 steps), increasing ligand rotamer rotations (rot_r 0.5), and omitting H1R for ANM calculations (lanmanm omit_no) since induced-fit refinement of histamine was focused on Ra-HBP2. Spawning was performed only for the second histamine within 8 Å of the COM of Ra-HBP2 (spawn point # xyz within 8.0).

### Analyses

The COMs were calculated using Chimera [23]. Histamine exploration trajectories produced by the PELE algorithm were viewed, clustered and analyzed using the Visual Molecular Dynamics (VMD) program [24]. The last ~25000 trajectories for each simulation were used for analyses since the first 100 trajectories in PELE are reaching a stable energy state. The VMD cluster analyses for the simulations were performed every 10 trajectories with a 5 Å cutoff distance between histamine trajectories to compute 10 color-coded histamine clusters (ordered according to number of trajectories per histamine cluster). This cut-off criterion was due to the number of trajectories (out a total of ~25000 trajectories) and to depict histamine exploration and preference by forming dense clusters (5 Å cutoff distance) near the COMs of either protein. Graphs were plotted using GNUPLOT.

## Results

### Histamine dynamics between the human H1R and the tick Ra-HBP2

**Figure 1:**
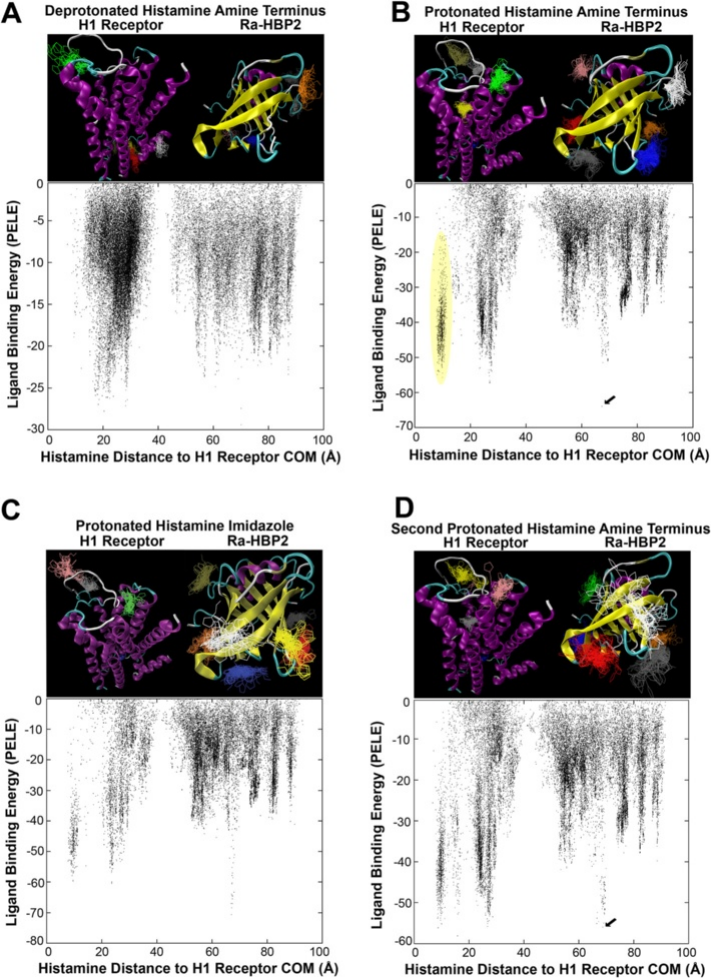
**Competitive histamine binding between the human H1R and the tick Ra-HBP.** The top panels correspond to the results in Table 1 from the VMD cluster analyses (see Analyses section in Methods for the cut-off criteria). The histamine color-coded clusters surrounding the protein structures (H1R, left – magenta; Ra-HBP2, right – yellow) are ordered according to number of trajectories within that specific histamine cluster as blue>red>darkgrey>orange>yellow>brown>grey>green>white>pink. (Angles of the protein structures were adjusted as best as possible to visualize the histamine clusters, but some clusters in the background may be difficult to fully see.) The bottom panels depict a scatter plot of histamine trajectories according to its distance (in Å) from the COM of the H1R (x-axis) and its respective histamine binding energy in kcal/mol (y-axis; as calculated by PELE) for the deprotonated histamine **(A)**, protonated amine terminus of histamine **(B)**, protonated nitrogen of the imidazole of histamine **(C)**, and the second protonated amine terminus histamine migration simulation **(D)** using the bound trajectory indicated by an arrow in **(B)**. The distance between the COM of H1R and its active site is 5 Å-10 Å. The region circled in yellow of scatter plot **(B)** indicates the yellow histamine cluster near the H1R active site (top panel) – the distance of this cluster is ~10 Å from the COM of H1R.

Figure 1 clearly depicts that simultaneously using the human H1R and the tick Ra-HBP2 for histamine exploration causes the data to split into two main clusters – the midpoint being the starting position of histamine at 42 Å equidistant from the COMs of both proteins. These two main clusters are also clearly distributed within their respective COMs (Additional file 1). The relative binding energies also suggests a different histamine exploration between the two proteins (Figure 1A).

Protonation states of ligands (and proteins) may impact protein-ligand interactions. Adding a hydrogen ion at the amine terminus of histamine (protonated) in the protein competition simulation causes a greater exploration around the COM of Ra-HBP2 (Figure 1B) compared with the deprotonated amine terminus of histamine (Figure 1A). Additionally, the protonated amine terminus causes an equal span of exploration (~40 Å) for both proteins (Figure 1B) than the deprotonated (Figure 1A). Further protonating the nitrogen atom in the imidazole of histamine causes less exploration for the H1R compared with the two other protonated states (Figure 1C and Additional file 1C). Therefore, as histamine increases its protonated state it reduces its exploration of the human H1R when the tick Ra-HBP2 is present (Figure 1A-C and Additional file 1A-C).

The VMD cluster analyses are structurally depicted above their respective data points in the scatter plots of Figure 1. The H1R is a transmembrane GPCR that spans the lipid bilayer (cell membrane) possessing an extracellular region, a membrane region and a cytoplasmic region [25]. The deprotonated histamine amine terminus seems to explore more the intracellular region (top panel Figure 1A, histamine clusters red, yellow, brown and white), which may be unlikely since (in response to tissue damage) the H1R is activated by extracellular histamine [1]; therefore, it must initially cross the lipid bilayer. The protonated amine terminus, however, produces a more realistic scenario. According to these simulations, adequate histamine binding for the H1R seems to rely on the protonated amine terminus of histamine since it clusters in proximity to the extracellular region and the H1R active site – located ~10 Å from its COM (the yellow histamine cluster in the structural representation coincide with the yellow circled data points in the scatter plot of Figure 1B). The crystal structure of the H1R (PDB: 3RZE) has its ligand positioned ~9 Å from the COM. This additional H1R active site exploration can be seen in all simulations except for the deprotonated state of histamine (Figure 1 and Additional file 1). The cluster analysis for the protonated imidazole of histamine shows an increased exploration for Ra-HBP2 and, although it explored in proximity of the H1R active site (as depicted in the scatter plot of Figure 1C), it did not meet the criteria for the cluster analysis (as depicted by the missing active site cluster in the structural representation of Figure 1C). Deprotonated histamine also produces less favorable binding energies (−30 kcal/mol; Figure 1A) compared with the two other protonated states (Figure 1B, −70 kcal/mol; Figure 1C, −80 kcal/mol).

For the native histamine position in the crystal structures of the tick Ra-HBP2, the COM of the first histamine (PDB: 1QFV) has a distance of 7.5 Å from the COM of the Ra-HBP2 and the COM of the second histamine (PDB: 1QFT) has a distance of 4.5 Å from the COM of the Ra-HBP2. To investigate how the second histamine will explore the two proteins, once the first histamine is bound to the Ra-HBP2, a subsequent PELE histamine exploration was performed using the lowest energy trajectory that was farthest from the COM of the H1R (indicated by the arrow in Figure 1B). This trajectory for the first histamine had a distance of 8.5 Å from the COM of Ra-HBP2 (a root mean square deviation, RMSD, of 1 Å from its native position). The lowest energy that was farthest from the COM of H1R for the second histamine exploration (indicated by the arrow in Figure 1D) reached a distance of 9.5 Å from the COM of Ra-HBP2 (a RMSD of 5 Å from its native position).

Overall, the cluster analyses revealed that both protonated states of histamine met the cut-off criterion by producing twice as many trajectories (~1,000) than the deprotonated state (~500 trajectories). Table 1 shows the specific number of trajectories per histamine cluster per protein and that all histamine protonation states show a preference to explore the Ra-HBP2 (deprotonated = 64%; protonated amine terminus = 69%; protonated imidazole = 83% – percentages were calculated as Ra-HBP2 clusters divided by sum total of clusters). Only the protonated amine terminus histamine was used for all subsequent simulations (simply referred to as histamine, henceforth) since it also explored and formed clusters within the H1R active site (i.e., the null hypothesis). Although slight, Figure 1D (and Table 1) shows that there is an increase in Ra-HBP2 preference for the second histamine (75%) compared with the first histamine (69%).

**Table 1:** **Number of histamine trajectories per cluster**

**Histamine Cluster**	**Blue**	**Red**	**Darkgrey**	**Orange**	**Yellow**	**Brown**	**Grey**	**Green**	**White**	**Pink**	**Total**
**Run/Protein**											
**Deprotonated**											
H1R	-	63	-	-	40	37	-	35	32	-	207
Ra-HBP2	210	-	44	41	-	-	35	-	-	30	360
**Sum Total**											**567**
**Protonated Amine Terminus**											
H1R	-	-	-	-	105*	101	74	71	-	-	351
Ra-HBP2	210	181	131	117	-	-	-	-	68	68	775
**Sum Total**											**1126**
**Protonated Imidazole**											
H1R	-	-	-	-	-	-	58	53	-	51	162
Ra-HBP2	163	153	126	125	91	74	-	-	53	-	785
**Sum Total**											**947**
**2** ^**nd**^ **Histamine**											
H1R	-	-	-	-	126	-	77*	-	-	71	274
Ra-HBP2	170	144	140	132	-	78	-	74	74	-	812
**Sum Total**											**1086**

The colors correspond to the number of trajectories for each color-coded histamine cluster in the top panels for the simulation runs in Figures 1A (Deprotonated), 1B (Protonated Amine Terminus), 1C (Protonated Imidazole) and 1D (2^nd^ Histamine). The color-coded histamine clusters were produced by VMD to depict protein preference for histamine exploration. The asterisk (*) denotes the color-coded histamine clusters in Figures 1B (Protonated Amine Terminus) and 1D (2^nd^ Histamine) that reached within 10 Å of the H1R active site. The numbers in boldface represent the sum total of trajectories of H1R and Ra-HBP2.

The crystal structure of Ra-HBP2 is protonated at Asp24/Asp110 (PDB: 1QFT and 1QFV). The Asp73/Asp124 of the H1R are highly conserved among the Class A GPCRs and are also protonated upon activation, i.e., upon histamine binding [10]. The simulations thus far have explored the protonated state of the Ra-HBP2 and the deprotonated state of the H1R. Additional runs were performed using different protonation states of both proteins (and combinations thereof), since protonation of specific residues (e.g., Asp) may also impact protein-ligand interactions. Additional file 1E shows that the deprotonated state of the Ra-HBP2 and the protonated state of the H1R showed a higher preference for the Ra-HBP2 – similar to the protonated imidazole (Additional file 1C). The remaining altered protonated states, however, did not alter the preference for histamine to explore the Ra-HBP2 (Additional file 1D and F).

### The Ra-HBP2 lipocalin impedes histamine exploration of the H1R active site

**Figure 2:**
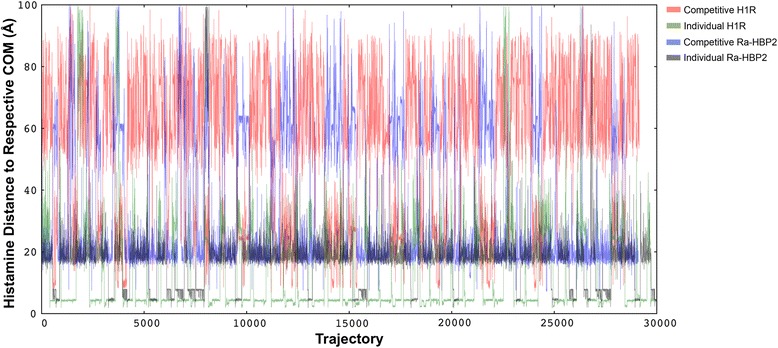
**Histamine exploration using both proteins separately.** The panel depicts the COM distance (y-axis) for each respective protein along their trajectories (x-axis) during competitive (red and blue lines) and individual (green and grey lines) histamine migration simulations. There are more trajectories for the individual runs since it is more computationally expensive for the PELE algorithm to simulate ligand migration for both proteins simultaneously.

Histamine exploration simulations were also performed using either protein separately to test for any bias towards conformation of the protein structures (inactive or active) and to test whether histamine can sufficiently sample the active site of either protein. Figure 2 shows histamine explores in close proximity to the COM of the H1R (~4 Å; green lines) and sufficiently samples the H1R active site(s). The H1R also binds two histamines with different affinities [26], so it seems that the simulations until now show that the Ra-HBP2 may impede the full H1R active site exploration for histamine since it is greatly reduced in the presence of the Ra-HBP2 (Figure 2; red lines). Histamine exploration of the Ra-HBP2 was similar for both simulations (15 Å-25 Å from the COM), but for the individual binding simulation histamine did explore the Ra-HBP2 active site (~4 Å from the COM; grey lines) more frequently than in the competitive binding simulation (blue lines).

Ticks secrete in their saliva a vast number of different protein families that counteract host defense mechanisms for successful blood feeding [27]. Among these protein families are Kunitz proteins that possess anti-coagulant and anti-inflammatory properties. To validate the competitive histamine-binding simulation, the tick-derived protease inhibitor (TdPI), a Kunitz salivary protein from the same tick species – *R. appendiculatus* [28] – was used as a false positive control. Additional file 2 shows that histamine explored the H1R more than TdPI with a high density at the H1R active site (~10 Å from the COM).

**Figure 3:**
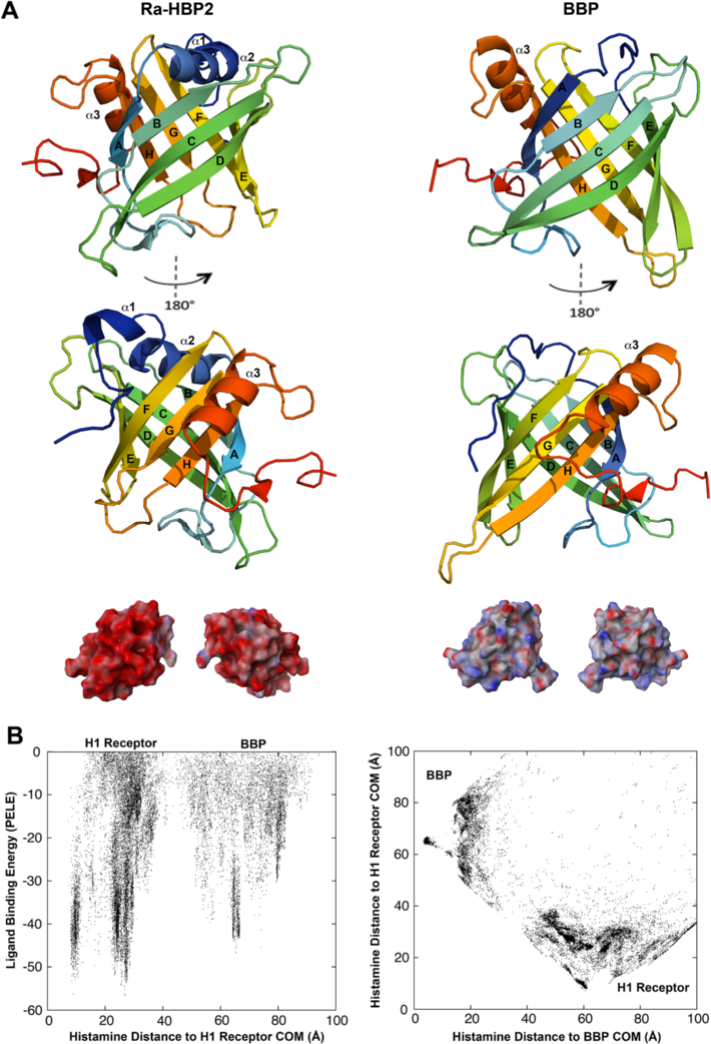
**The butterfly lipocalin (BBP) crystal structure was used as a negative control.** The tertiary structures for the Ra-HBP2 and the butterfly BBP (PDB: 1BBP) with 180° turn (below) are depicted in panel **(A)** and colored from the N-terminus (blue) to the C-terminus (red). The electrostatic potential below each tertiary structure in 180° turns (blue = positive, red = negative and white = neutral) was calculated according to the Poisson-Boltzmann equation implemented in the Schrodinger’s Maestro package. Panel **(B)** depicts the same respective Cartesian coordinates as in Figure 1 and Additional file 1 for competitive histamine binding between the human H1R and the BBP.

The ability to attract histamine may be due to the acidic net charge of the lipocalin, Ra-HBP2. To test this hypothesis, an initial histamine exploration was performed using the butterfly lipocalin, bilin-binding protein (BBP; PDB: 1BBP), from *Pieris brassicae*, and the human H1R. As reported by Paesen *et al.* [6], the BBP shares high structural similarities with the Ra-HBP2, but only 9% of the residues are identical. The electrostatic potential compared with the BBP shows that the surface of Ra-HBP2 is highly acidic (Figure 3A). These simulations show that although histamine did explore the butterfly BBP (Figure 3B) it was less than for the tick Ra-HBP2 (Figure 1B and Additional file 1B) and there was a greater preference for the human H1R (Figure 3B).

### Induced-fit refinement reveals insights on the dynamics of functional residues

**Figure 4:**
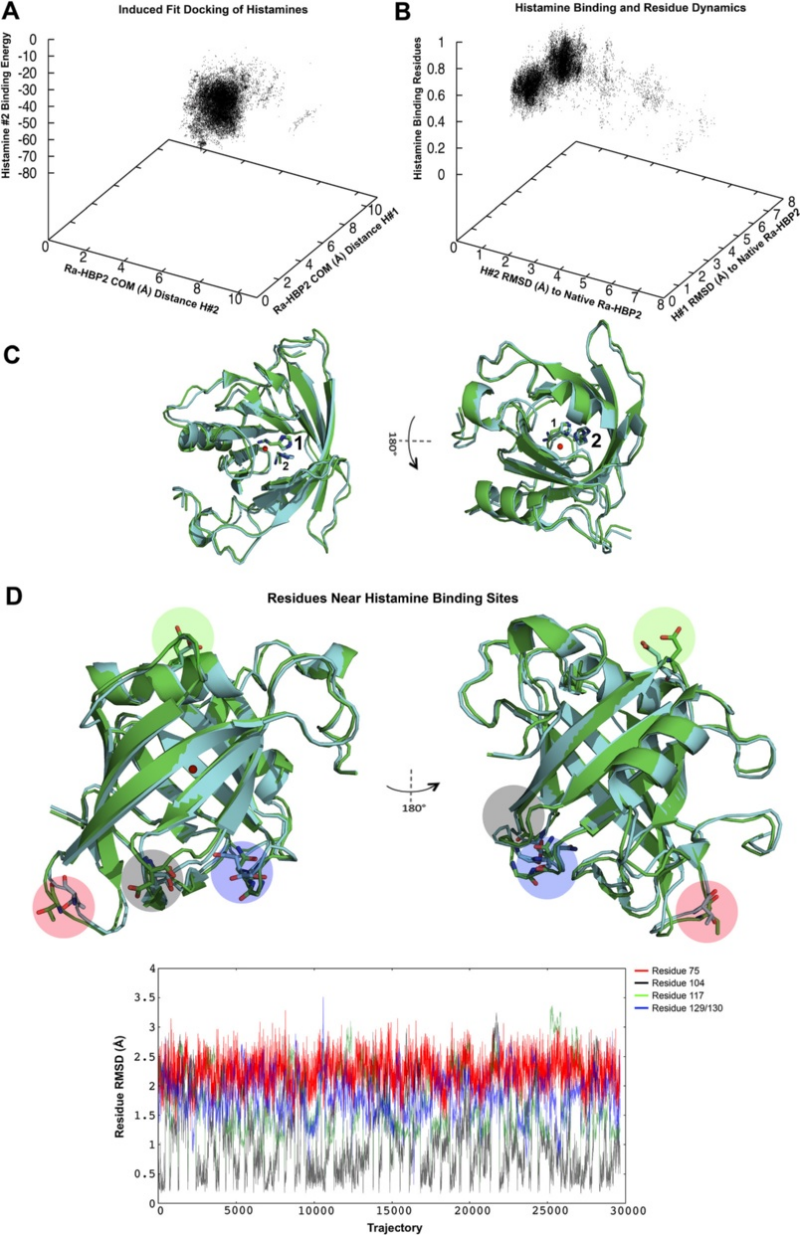
**Induced-fit refinement of the double histamine bound to Ra-HBP2.** The induced-fit refinement 3D plot **(A)** shows the COM distance to Ra-HBP2 for the first (y-axis) and the second histamine (x-axis) compared to the binding energy (z-axis) of the second histamine. Panel **B** depicts the RMSD to the native Ra-HBP2 (PDB: 1QFT) for the first (y-axis) and the second histamine (x-axis) compared to the side chain perturbations for the functional residues reported in [6] (z-axis). The open and closed mouths are respectively shown in 180° turn **(C)** with the backbone superposition of the Ra-HBP2 original conformation (green; PDB: 1QFT) and a trajectory <1.5 Å RMSD for both histamines (cyan) after induced-fit refinement. Panel **D** is the same superposition **(C)** in 180° depicting specific residues within β-hairpin loops of both ends of the lipocalin barrel with large side chain perturbations as graphically shown along their trajectories (color-coded respectively to the structural representation). The COM for Ra-HBP2 is shown as a red sphere.

The first histamine-binding site of Ra-HBP2 (PDB: 1QFV) is at the open mouth of the barrel occluded by β-hairpin loops formed by β-strands A-B (residues 34–50, also referred to as the ‘tongue’ loop that is structurally heterogeneous among lipocalins [6]), C-D (residues 69–78), E-F (residues 102–105) and G-H (residues 124–133). The second histamine-binding site is located at the closed mouth of the barrel, near the N-terminus of the Ra-HBP2 – a region occluded by the α-helix1, α-helix2 (α1 and α2; residues 17–26) and the β-hairpin loop formed by β-strands F-G (residues 116–119). (The positions for these loops and secondary structures are denoted in Figure 3A). A PELE ligand-binding refinement (or induced-fit refinement) was performed since the second histamine (Figure 1D) with the lowest energy, farthest from the COM of H1R, reached a distance of 9.5 Å (a RMSD of 5 Å from its native position). The data collected from the induced-fit refinement simulation shows that both histamines mainly explored distances within their respective native COMs from the COM of the Ra-HBP2 (Figure 4A) and that all atoms (excluding hydrogen atoms) for each histamine reached a RMSD of <1.5 Å from their native positions (PDB: 1QFT; Figure 4B).

The reported histamine interacting residues of the Ra-HBP2 ([17] residues as reported in reference [6]) were analyzed to assess any side chain conformational changes during induced-fit refinement. A backbone alignment of the Ra-HBP2 to its native structure (PDB: 1QFT) showed that these residues averaged <1 Å RMSD from the native structure (Figure 4B) and that the trajectories for each individual residue had a RMSD range of 0 Å-1.9 Å (Additional file 3). As shown in Figure 4A, induced-fit refinement caused both histamines to explore within their respective COMs, e.g., where the first histamine was closest to the COM of its native structure (7.5 Å), also had the second histamine closest to the COM of its native conformation (4.5 Å) (the green structure in Figure 4C represents the original conformation of the Ra-HBP2 and both histamine positions). As seen by the structural representation in Figure 4C the first histamine was bound towards the open mouth of the Ra-HBP2, while the second histamine was bound on the opposite end (the closed mouth of the barrel). Visual inspection of the induced-fit refinement trajectories revealed that the β-hairpin loops at both ends of the Ra-HBP2 barrel had specific residues with large conformational changes (Figure 4D). Except for Asp117, these residues are in close proximity to the Ra-HBP2 clusters depicted above the scatter plots in Figure 1B-D.

### Ra-HBP2 may bind more than two histamine molecules

**Figure 5:**
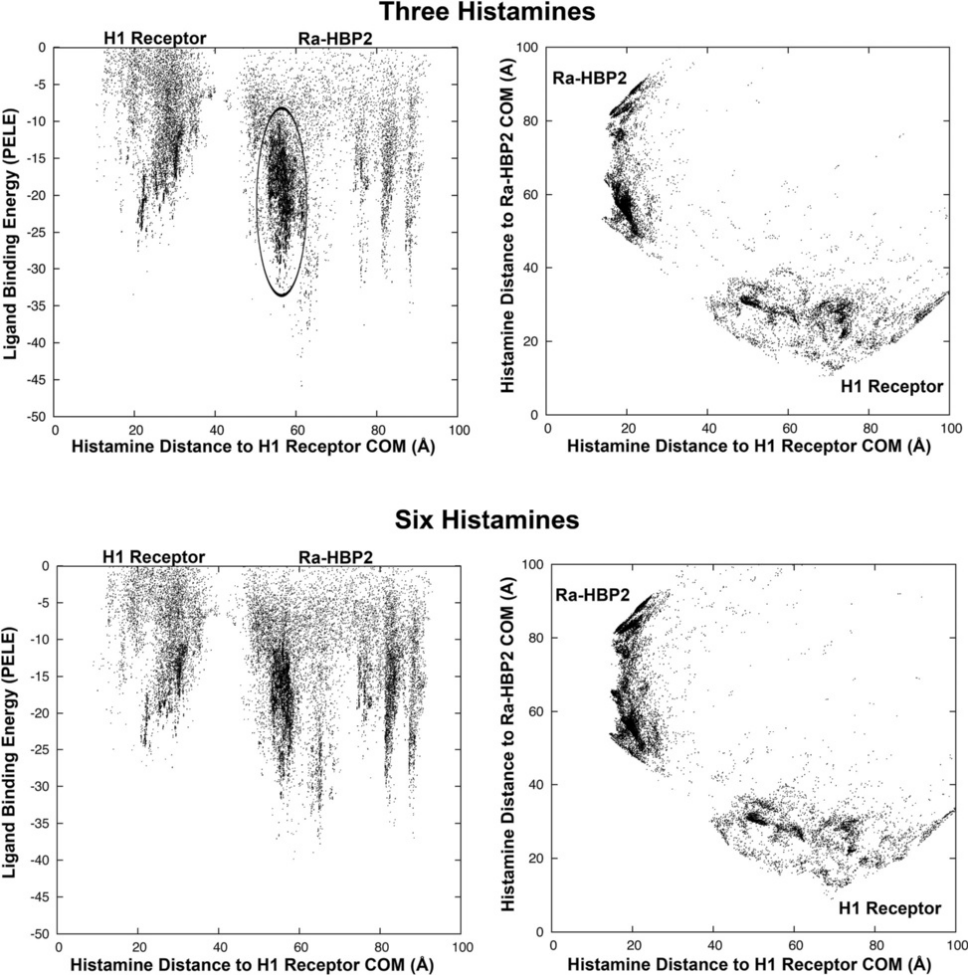
**Additional histamine does not explore the H1R active site in the presence of the double bound Ra-HBP2.** The panels on the left depict the histamine trajectories according to its distance (Å) from the COM of the H1R (x-axis) and its respective histamine PELE binding energy. The panels on the right depict the histamine trajectories according to its distance from the COM of the tick Ra-HBP (x-axis) and the human H1R (y-axis). The circled cluster is within the trajectories of the third histamine binding site(s) represented in Figure 6.

Once Ra-HBP2 has two histamines bound to it, will additional histamines explore the H1R more? To answer this question another histamine was added to explore the double bound Ra-HBP2 structure after induced-fit refinement (the cyan structure in Figure 4). This additional histamine did not explore the H1R active site as noted by the missing cluster in Figure 5; similar to the deprotonated amine terminus of histamine described above (Figure 1A and Additional file 1A and G). The trajectory of the third histamine (chosen from within the cluster circled in Figure 5) is at a distance of ~19 Å from the COM of the Ra-HBP2 at the open mouth of the barrel sandwiched between carboxyl-terminus and β-hairpin loops A-B (the ‘tongue’ loop) and C-D (Figure 3). Simulations of up to six histamines (Figure 5) still did not explore the H1R active site.

Thus far, there seems to be a slight increase in histamine exploration of the bound Ra-HBP2 with a reduced exploration of the H1R active site (Figures 1D and 5). This simulated attraction towards the bound Ra-HBP2 suggests ligand-binding cooperativity, i.e., the binding of one ligand to a protein affects the affinity of subsequent ligands for the same protein (and, in this case, a competitive protein). It will be, however, far too speculative at this point to continue without further experimental data for proof of principle since previous kinetic measurements were unable to determine cooperativity due to insufficient labeling of subsequent binding sites of the Ra-HBP2 [6].

## Discussion

The simulations presented here depict, for the first time, a competition at the host-tick interface for histamine binding between its native target, the human H1R, and the secreted tick salivary lipocalin, Ra-HBP2. The competitive binding simulations show that histamine explores the Ra-HBP2 more than the H1R and the more Ra-HBP2 sequesters histamine the less it explores the H1R active site. The data presented here therefore provides insight for the host-tick interface; specifically, to explain the dynamics of how salivary lipocalins allow the tick to evade host inflammatory responses to imbibe a blood meal.

Circulating histamine also causes vasodilation, a physiological response necessary for ticks to successfully obtain a blood meal [27]. How can histamine be circulating around the injury site without binding to its native receptor (e.g., H1R), thus causing an inflammatory response? The attraction towards the Ra-HBP2 seems to depend on the protonated state of histamine with an increasing exploration as deprotonated < protonated amine terminus < protonated imidazole (Figure 1A-C). This implies that during tick feeding, histamine may need to be protonated for Ra-HBP2 to efficiently sequester it in order to counteract inflammation. Under physiological conditions, histamine is usually protonated at the amine terminus (with a pKa of ~9.75), while the imidazole will not be protonated (pKa of ~6). Interestingly, tick saliva is basic (pH 9.0-9.5) and this has been attributed to one of the mechanisms pathogens hitchhike on to infect the host [29]. Additionally, there is a high hydrogen ion concentration during the course of an inflammatory response – as seen in rheumatoid arthritis patients [30]. Therefore, the preference of histamine for Ra-HBP2 may be facilitated by the basic properties of tick saliva by neutralizing or fluctuating the pH at the injury site.

**Figure 6:**
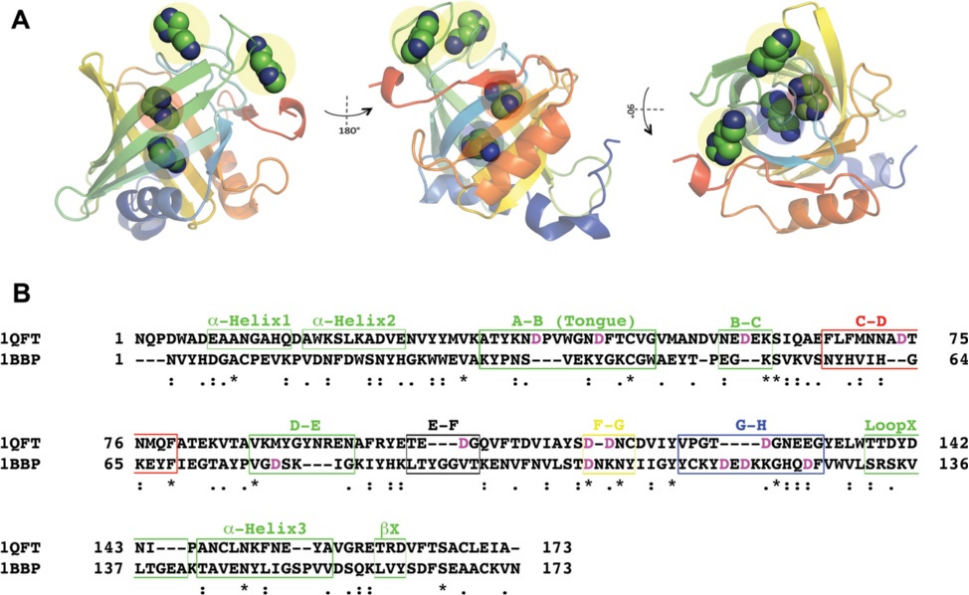
**Positions of other histamine-binding sites and alignment of the tick Ra-HBP2 (PDB: 1QFT) and the butterfly BBP (PDB: 1BBP).** The representative predicted positions **(A)** for the third histamine-binding site (encircled yellow) while bound to the first (red) and second histamine (blue) to the Ra-HBP2 (color-coded from N-terminus (blue) to C-terminus (red)). The positions of the third histamine are within ~12 Å distance from each other – this is also the same span of exploration represented in the encircled cluster of Figure 5. The pairwise alignment **(B)** shows positions of the α-helices and β-hairpin loops of the Ra-HBP2 (boxed) in relation the BBP. Boxes of different color (i.e., red, black, yellow and blue) correspond to the same colors indicated in Figure 2D. Asp residues within the loop regions are in magenta. The (.) denotes similar residues and the (*) denote identical residues.

Compared with other members of the lipocalin protein family, the closed mouth of the Ra-HBP2 barrel is unusually blocked by α-helix1, α-helix2 and the β-hairpin loop F-G (residues 116–119) – the α-helix2 is missing from other lipocalins [6]. As shown in Figure 4D, the Asp117 residue of this loop undergoes large conformational changes during the induced-fit refinement of the second histamine. The β-hairpin loops occluding the open mouth of the Ra-HBP2 also showed specific residues with large conformational changes and this region is represented as the large histamine exploration cluster for the Ra-HBP2 encircled in Figure 5. The predicted sites for the third histamine shown in Figure 6A are within this cluster. These β-hairpin loops (at both ends of the lipocalin barrel) were reported to have a functional role in sequestering histamine [6] and the results in the current study concur with this hypothesis. It is worth noting that these loops of the Ra-HBP2 possess more Asp residues than the butterfly lipocalin BBP (except for loop G-H; Figure 6B) and these acidic residues may be responsible for lack of histamine exploration of the H1R active site in the presence of the double histamine bound tick Ra-HBP2 (Figure 6).

## Conclusions

Tick salivary glands has been of major focus for high-throughput sequencing due to their role in the transmission of tick-borne pathogens [31] and an emerging topic in vector biology is to reveal how tick salivary proteins facilitate blood feeding, and their role as determinants of vector capacity [32]. As obligate hematophagous arthropods, ticks contain, in their saliva, an arsenal of macromolecules that counteract host defense mechanisms in order to obtain a blood meal (revised in [27]), thereby, indirectly providing a gateway for pathogen transmission. Providing evidence and insight, at the atomic level, on the counteraction of host defense mechanisms by tick salivary proteins will increase our knowledge on what precisely happens at the interface of the host-ectoparasite interaction. This evidence and insight may also explain the pathogenesis that ticks facilitate. Additionally, one of the most challenging subjects in pharmacology is drug target specificity (or pharmacological promiscuity) and to refine drugs for specificity is experimentally expensive. Therefore, the computational methods presented here may also assist in a rational approach for drug design, including the dynamics of competitive ligand binding.

## Additional files

Additional file 1:
**Histamine migration for both proteins and their protonation states.** All panels depict a scatter plot of the histamine trajectories according to its distance from the COM of the tick Ra-HBP (x-axis) and the human H1R (y-axis). Panel (A-C) and (H) are the respective representatives for the scatter plots in Figure 1A-D. The circled points in panel (B) indicate the clusters of histamine that explore near the H1R active site. Panels (D-F) are the different protonated states for the aspartic acid residues of the H1R (Asp73 and Asp124) and the Ra-HBP (Asp73 and Asp124). Panel (G) shows that the deprotonated state for the second histamine exploration resembles that of (A). Additional file 2:
**The tick salivary TdPI crystal structure was used as a false positive control.** The panels depict the same respective Cartesian coordinates as in Figure 1 and Additional file 1 for competitive histamine binding between the human H1R and the Kunitz salivary peptide (TdPI; PDB: 2UUX) from *R. appendiculatus*. Additional file 3:
**Conformational changes for the tick Ra-HBP2 residues reported by [** 6 **] known to interact with histamine.** The RMSD frequency for each interacting residue (y-axis) for the trajectories (x-axis) produced during induced-fit refinement of the double histamine bound Ra-HBP2.

## Abbreviations

ANM: Anisotropic network model
BBP: Bilin-binding protein
COM: Center of mass
CPUs: Computer processing units
GPCR: G-protein coupled receptor
H1R: Human histamine 1 receptor
OPLS: Optimized potentials for liquid simulations
PELE: Protein Energy Landscape Exploration
Ra-HBP2: *Rhipicephalus appendiculatus* histamine-binding 2 protein
RMSD: Root mean square deviation
TdPI: Tick-derived protease inhibitor
