# Supplementary figures and images for: Antihistamine response: a dynamically refined function at the host-tick interface
Source: Parasit Vectors. 2014 Oct 31;7:491. doi: 10.1186/s13071-014-0491-9 (PMC4226919; doi:10.1186/s13071-014-0491-9)

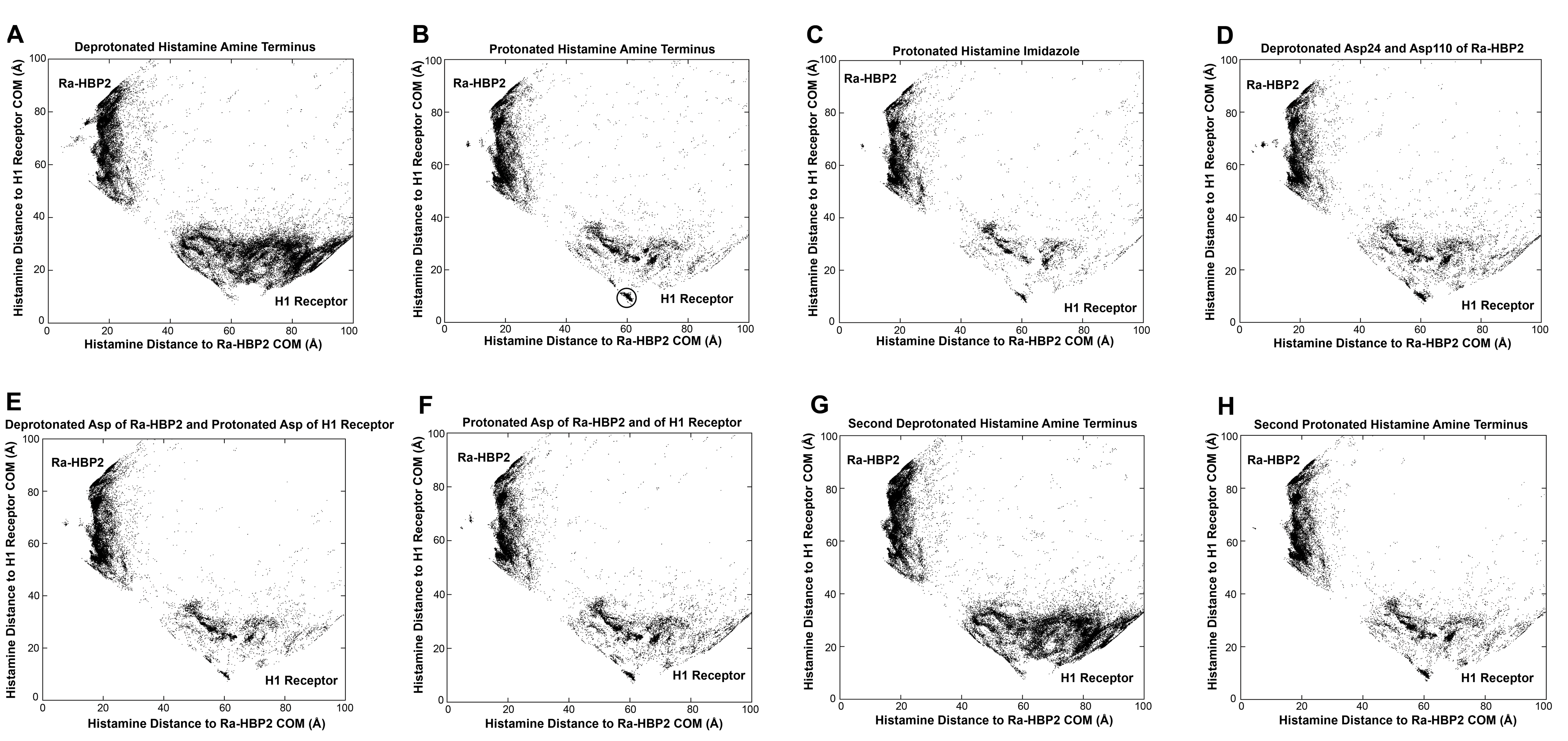

Supplement: Additional file 1: — Histamine migration for both proteins and their protonation states. All panels depict a scatter plot of the histamine trajectories according to its distance from the COM of the tick Ra-HBP (x-axis) and the human H1R (y-axis). Panel (A-C) and (H) are the respective representatives for the scatter plots in Figure 1A-D. The circled points in panel (B) indicate the clusters of histamine that explore near the H1R active site. Panels (D-F) are the different protonated states for the aspartic acid residues of the H1R (Asp73 and Asp124) and the Ra-HBP (Asp73 and Asp124). Panel (G) shows that the deprotonated state for the second histamine exploration resembles that of (A). [file 13071_2014_491_MOESM1_ESM.tiff]

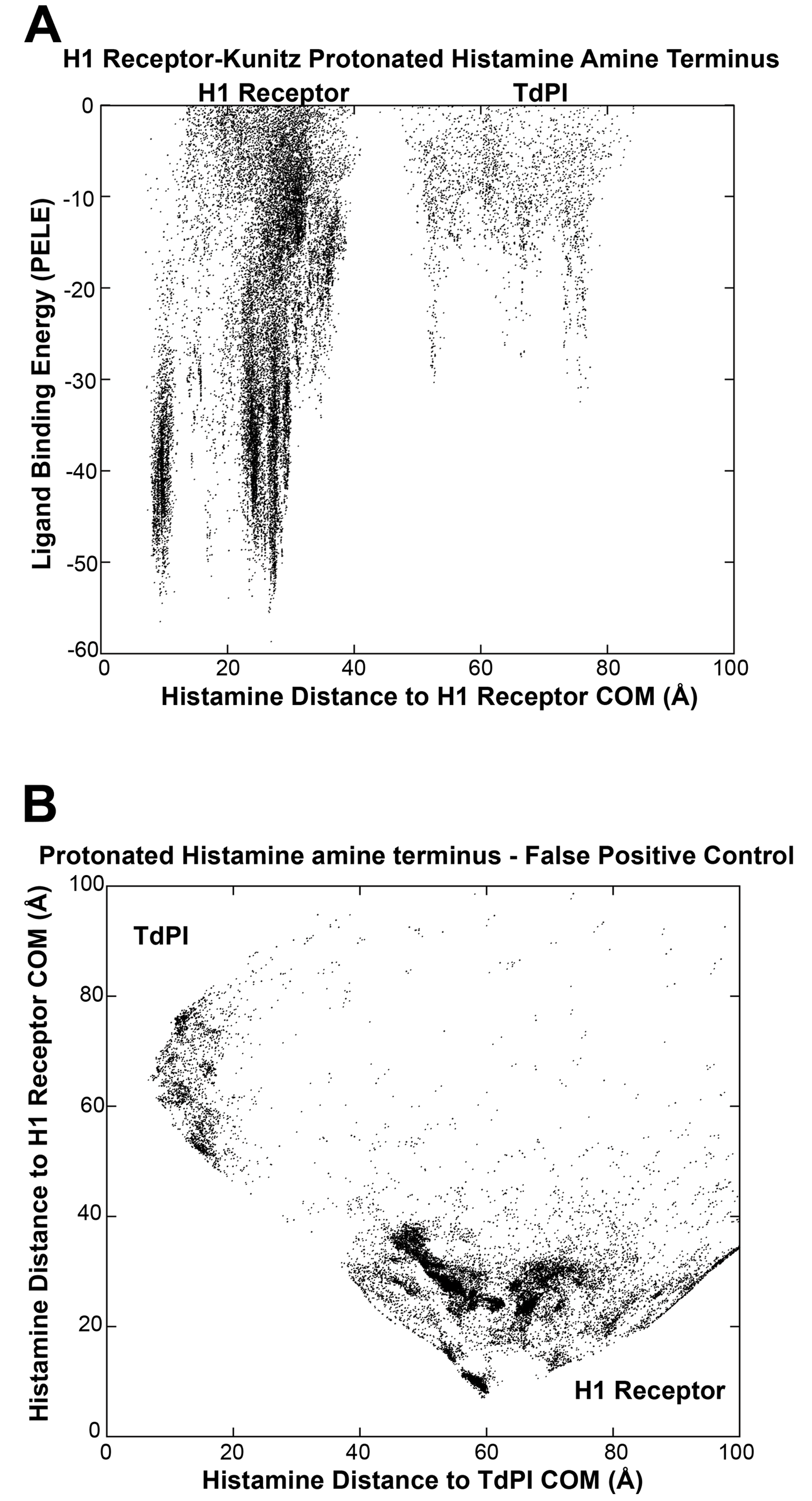

Supplement: Additional file 2: — The tick salivary TdPI crystal structure was used as a false positive control. The panels depict the same respective Cartesian coordinates as in Figure 1 and Additional file 1 for competitive histamine binding between the human H1R and the Kunitz salivary peptide (TdPI; PDB: 2UUX) from R. appendiculatus. [file 13071_2014_491_MOESM2_ESM.tiff]

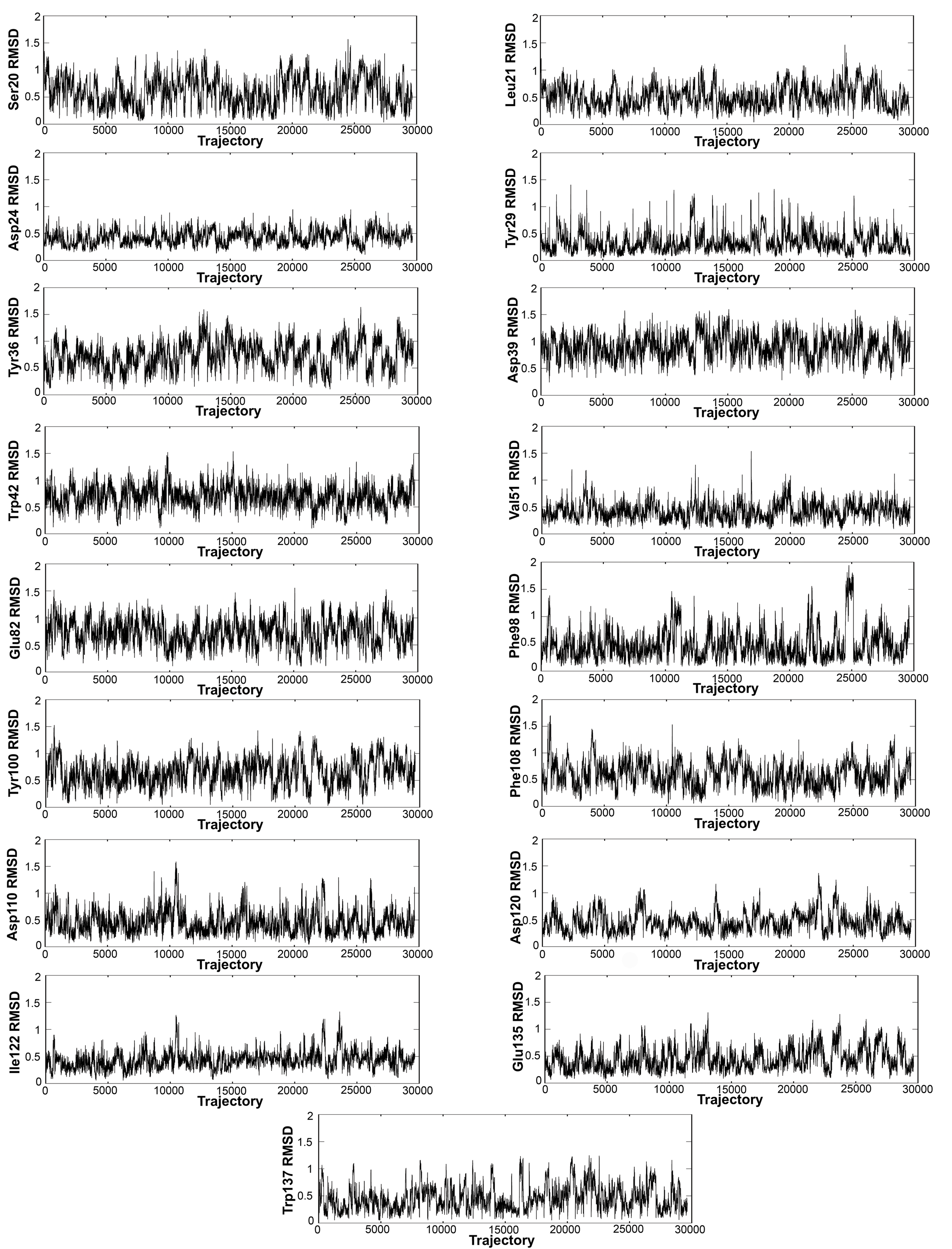

Supplement: Additional file 3: — Conformational changes for the tick Ra-HBP2 residues reported by [ 6 ] known to interact with histamine. The RMSD frequency for each interacting residue (y-axis) for the trajectories (x-axis) produced during induced-fit refinement of the double histamine bound Ra-HBP2. [file 13071_2014_491_MOESM3_ESM.tiff]
